# Clopidogrel treatment may associate with worsening of endothelial function and development of new digital ulcers in patients with systemic sclerosis: results from an open label, proof of concept study

**DOI:** 10.1186/s12891-016-1072-1

**Published:** 2016-05-17

**Authors:** Konstantinos Ntelis, Vasileios Gkizas, Alexandra Filippopoulou, Periclis Davlouros, Dimitrios Alexopoulos, Andrew P. Andonopoulos, Dimitrios Daoussis

**Affiliations:** Division of Rheumatology, Department of Internal Medicine, Patras University Hospital, University of Patras Medical School, 26504 Rion, Patras, Greece; Department of Cardiology, Patras University Hospital, University of Patras Medical School, 26504 Rion, Patras, Greece

**Keywords:** Systemic sclerosis, Scleroderma, Clopidogrel, Digital ulcers, Platelets

## Abstract

**Background:**

Activated platelets release serotonin that binds 5-HT_2B_ receptor on fibroblasts leading to fibroblast activation. Clopidogrel, an inhibitor of ADP-dependent platelet activation prevents fibrosis in animal models of systemic sclerosis (SSc). We aimed at assessing whether i) ADP-dependent platelet activation is increased in patients with SSc compared to healthy subjects and patients with rheumatoid arthritis (RA) and ii) whether clopidogrel can effectively suppress ADP-dependent activation, reduce circulating serotonin levels and hence, favorably affect fibrosis or vasculopathy in patients with systemic sclerosis.

**Methods:**

Thirteen patients with SSc were recruited. Platelet activation was assessed by aggregometry prior to and following 14 days of clopidogrel treatment. At the same time points serotonin and soluble vascular cell adhesion molecule 1 (s-VCAM1), a marker of endothelial dysfunction, were measured.

**Results:**

ADP-dependent platelet activation was similar between patients with SSc (*n* = 13), patients with RA (*n* = 28) and healthy subjects (*n* = 22) (mean ± SEM AU*min: 392.1 ± 58.4, 535.5 ± 61.33 and 570.9 ± 42.9 in patients with SSc, patients with RA and healthy subjects respectively, *p* = 0.14). Clopidogrel treatment significantly reduced platelet activation in patients with SSc (mean ± SEM AU*min: 392.1 ± 58.4 vs 163.8 ± 51.7, *p* = 0.014). Clopidogrel treatment did not affect serotonin levels but led to a significant increase in s-VCAM1 (*p* = 0.03). Three patients developed new digital ulcers during the study. The potential association of the study drug with the development of new digital ulcers led to early termination of the study.

**Conclusion:**

Clopidogrel may worsen markers of endothelial function and associate with development of new digital ulcers in patients with SSc.

**Clinical trial registration:**

ISRCTN63206606. Registered 02/Dec/2014.

## Background

Systemic Sclerosis (SSc) is a systemic rheumatic disease characterized by obliterative vasculopathy, autoimmunity and progressive fibrosis leading to tissue injury. The skin and several internal organs are involved during the disease course; involvement of vital organs leads to increased mortality and morbidity. To date, no approved disease-modifying therapies exist for the treatment of patients with SSc. Numerous experimental drugs or interventions have been investigated for the treatment of visceral fibrosis in systemic sclerosis, some of them demonstrating promising results [1–4]. However, the need for new therapeutic targets in SSc remains crucial.

The main pathogenetic model of SSc recognizes endothelial cell injury, immune activation and fibrosis as the key points in the disease process [5, 6]. Lately, accumulating evidence suggests that platelets are not just cell fragments regulating hemostasis, but may be active players in the pathogenesis of several inflammatory or autoimmune diseases, including SSc [7–9]. Evidence suggests that there may be increased platelet activity in patients with SSc, due to the underlying vascular damage [10, 11] and many platelet-derived molecules, such as beta-thromboglobulin, thromboxane B2 and platelet factor 4, have been reported to be elevated in patients with SSc [12, 13]. A recent study has provided evidence that serotonin (5-hydroxytryptamine [5-HT]) released in peripheral tissues upon activation of platelets may induce skin fibrosis through activation of fibroblasts via 5-HT_2B_ receptors [14]. These data point to the direction of a novel pathogenetic model of SSc where platelets and serotonin act as the link between endothelial dysfunction and fibrosis (endothelial dysfunction → platelet activation → serotonin release → fibroblast activation → fibrosis). Moreover, inhibition of platelet activity by clopidogrel treatment leads to a reduction of fibrosis in bleomycin-induced and tight skin murine models of scleroderma [14]. It is not known whether serotonin levels are increased in patients with SSc, but it has been found to be elevated in closely related conditions, such as Raynaud’s phenomenon and pulmonary hypertension [15, 16]. Antiplatelet agents, such as aspirin, which have been used previously in patients with SSc in order to improve vasculopathy have failed to show any efficacy [17]. So far, the effect of platelets inhibition by clopidogrel, a P2Y_12_ receptor antagonist, has not been investigated *in vivo* in patients with SSc. Clopidogrel is indicated for adult patients suffering from myocardial infarction or acute coronary syndrome, ischemic stroke or established peripheral arterial disease. Clopidogrel inhibits adenosine diphosphate (ADP) binding to its platelet P2Y_12_ receptor and subsequently the ADP-mediated activation of the glycoprotein GPIIb/IIIa complex, thereby inhibiting platelet aggregation. ADP-dependent platelet activation is one of the pathways which lead to platelet activation.

In this study we aimed to assess whether i) ADP-dependent platelet activation is increased in patients with SSc compared to healthy subjects and patients with rheumatoid arthritis (RA) and ii) whether clopidogrel can a) effectively suppress ADP-dependent activation of platelets, b) reduce circulating levels of serotonin, a significant profibrotic mediator and finally c) favorably affect fibrosis or vasculopathy in patients with systemic sclerosis. We report herein that clopidogrel effectively inhibits ADP-dependent activation of platelets but does not improve either fibrosis or endothelial function in patients with SSc. To the contrary, it may associate with a deterioration of disease course, triggering the development of new digital ulcers.

## Methods

### Patients

We enrolled 14 patients with a diagnosis of SSc, fulfilling the 2013 ACR/EULAR criteria for the classification of the disease [18]. One patient did not comply with treatment and was withdrawn from the study. Baseline demographic and clinical characteristics of the remaining 13 patients are presented in Table 1. Most patients were female (*n* = 12) with a mean age of 59.5 years and had limited disease (*n* = 8) with a mean disease duration of 10.9 years. All patients underwent a complete physical examination and a detailed review of their medical files prior to study enrolment. Other variables were also evaluated (full blood count, biochemistry profile, autoantibody profiles, electrocardiogram and cardiac ultrasound). All patients were Anti-Scl-70 (anti-topoisomerase I antibodies) or anti-ACA (anti-centromere antibodies) positive (*n* = 5 and *n* = 8 respectively) and had no change in medications administered during the last 6 months before enrollment. None of the study subjects was under any kind of antiplatelet treatment. No change in immunosuppressive treatment was allowed during the study. Exclusion criteria included a history of gastrointestinal or cerebral bleeding. ADP-dependent platelet activation was also measured in 28 patients with RA and 22 healthy subjects for control purposes. Most patients with RA were female (75 %) with a mean age of 59 years and a mean DAS28 of 2.9; no patient was receiving any antiplatelet agent. The majority of the patients with RA were on treatment only with synthetic DMARDs (71.4 %), mainly methotrexate, while the rest of them were receiving also a biologic DMARD (28.6 %). No patient was receiving drugs, such as antidepressants (including selective serotonin reuptake inhibitors, serotonin and noradrenaline reuptake inhibitors and tricyclic anti-depressants), any kind of anti-psycotic agent, anti-migraine agents (triptans), anti-convulsants, anti-parkinsonian agents, opioids and tramadol, that interfere with levels of serotonin. Healthy subjects were mostly female (90.9 %) with a mean age of 55 years and were not on any kind of antiplatelet or other treatment. APA and DD formed the Safety Board of the study and were responsible for evaluating all adverse events.

**Table 1:** Demographics, clinical characteristics and medications of study subjects

Patient no/sex/age in years	Disease Duration in years	Type of Disease	Organ Involvement	Concurrent medications
1/F/47	5	limited	GI	-
2/F/62	12	diffuse	Lung,PAH,GI	CYC
3/M/55	2	limited	Lung,GI	-
4/F/53	12	diffuse	Lung,DU,GI	MMF, Bos
5/F/53	12	limited	Lung,DU,GI	Pred, RTX, Bos
6/F/78	4	diffuse	Lung,DU,PAH,Musc	Pred, Bos, RTX
7/F/57	21	limited	Lung,DU,GI	Pred, Bos
8/F/58	3	diffuse	Lung,GI	RTX
9/F/66	9	limited	Lung,GI	MMF
10/F/59	17	limited	Musc	–
11/F/65	8	limited	Lung,GI	RTX
12/F/40	22	diffuse	Lung,GI,Musc	RTX
13/F/81	15	limited	GI	–

Pred: low dose prednisone; Bos: bosentan; MMF: mycophenolate mofetil; CYC: cyclophosphamide; RTX: rituximab

DU: digital ulcers; Musc: musculoskeletal involvement; GI: gastrointestinal involvement; PAH: pulmonary arterial hypertension

### Treatment

Patients with SSc were treated with the recommended dose of clopidogrel in everyday clinical practice (75 mg). Duration of the study had been planned to be 2 years. Finally, due to early discontinuation of the study, 12 patients completed 1 year of treatment, while one patient (#13) was treated with the study drug for 10 months.

### Indices of internal organ function

Standard PFTs (pulmonary function tests) were performed every 6 months during the study, including assessment of forced vital capacity (FVC) and diffusing capacity of carbon monoxide (DLco) corrected for hemoglobin concentration. PFT parameters are expressed as a percentage of normal predicted values. Estimated glomerular filtration rate (eGFR) was calculated at baseline and at 1 year with the 4 variables MDRD (Modification of Diet in Renal Disease Study Group) formula [19]. Complete cardiac ultrasound study was performed at baseline and at 12 months and right ventricular systolic pressure (RVSP) was determined.

### Overall functional impairment

Assessment of functional status was performed at baseline, 6 and 12 months using the modified for Scleroderma Health Assessment Questionnaire (SHAQ) [20].

### Clinical assessment of skin thickening

The modified Rodnan skin score (MRSS) [21, 22] was used for clinical assessment of skin thickening at baseline and at 1 year, by two experienced assessors (DD and KN).

### Levels of circulating serotonin and s-VCAM1

Whole blood was obtained from all participants at baseline and following 14 days of treatment. Platelet poor plasma was isolated from citrate tubes following a double centrifugation at 2000 g. Following the first centrifugation the top 75 % of plasma was removed with a plastic pipette, avoiding contamination, and underwent a second centrifugation at 2000 g. After the second centrifugation, the upper 75 % purified from the sedimented platelets, formed the platelet poor plasma. Samples from strenuous punctures were excluded. Serotonin was measured in these specimens with Radioimmunoassay (RIA) technique (DIAsource ImmunoAssays). Serum was also isolated at the same time points in order to evaluate the effect of treatment on the circulating levels of soluble vascular cell adhesion protein 1 (s-VCAM1), a marker of endothelial dysfunction [23]. Enzyme-linked immunosorbent assay (ELISA) technique was employed, using commercially available kit, according to the manufacturer’s instructions (R&D Systems, Minneapolis MN, USA). All samples were stored at -70 ° C.

### Assessment of ADP-dependent activation of platelets

The effect of treatment upon platelet activity was assessed through ADP-induced aggregation of platelets, measured by Multiplate Analyser (Roche), an instrument with five channels for parallel aggregometry measurements. Platelet activation was measured at baseline, following 14 days and 1 year of treatment. After 1:2 dilution of whole blood with 0.9 % NaCl solution and incubating for 3 min in the test cuvettes at 37 °C, the agonist (ADP) was added. Platelet aggregation was continuously recorded for 6 min. The adhesion and aggregation of platelets was measured by the change of electrical resistance between two sensor wires. Impedance is transformed to arbitrary aggregation units (AU) that are plotted against time (AU*min) [24]. Resistance to clopidogrel inhibition was defined as a AU*min value ≥468 on treatment [25].

### Statistical analysis

Statistical analysis was performed using the GraphPad Prism software version 5. All variables were tested for normality using D'Agostino's test. Data are presented as mean ± SEM, median or percentages, as appropriate. The paired Student’s t-test and Wilcoxon matched pairs test, were used as indicated, for normal and non-normal distributions respectively. One-way ANOVA was used for group comparisons. Unpaired t-test was used to compare unpaired values. Correlations between ADP-dependent platelet activation and other variables were analyzed by Pearson or Spearman test, as appropriate. Values of *p* < 0.05 were considered as statistically significant.

## Results

### No evidence of increased ADP-dependent platelet activation in patients with SSc

**Fig. 1:**
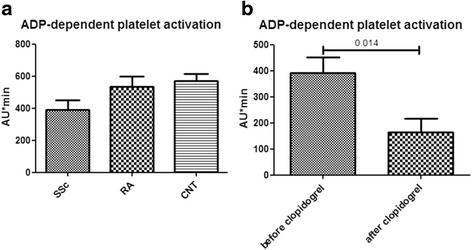
ADP-dependent platelet activation in patients with SSc is not increased compared to patients with RA and healthy subjects (**a**). Clopidogrel effectively inhibits ADP-dependent platelet activation in patients with SSc (**b**)

We first evaluated the ADP-dependent activation of platelets in patients with SSc compared to patients with RA and healthy subjects. No statistically significant differences were found between patients with SSc (*n* = 13), patients with RA (*n* = 28) and healthy subjects (*n* = 22) (mean ± SEM AU*min: 392.1 ± 58.4, 535.5 ± 61.33 and 570.9 ± 42.9 in patients with SSc, patients with RA and healthy subjects respectively, *p* = 0.14). These data are diagrammatically depicted in Fig. 1a.

Since many patients in our cohort had long standing disease we next sought to explore whether ADP-dependent platelet activation in patients with SSc correlates with disease duration. We found that ADP-dependent platelet activation in patients with early disease (as defined by disease duration ≤ 4 years, n = 3) was similar to that of patients with long standing disease (mean ± SEM AU*min: 315.7 ± 105.4 vs 415.0 ± 70.1 in patients with early vs late disease, respectively, *p* = 0.49). Moreover, no significant association of ADP-dependent platelet activation with age, gender, PFTs or MRSS in patients with SSc was found.

These data indicate that ADP-dependent platelet activation is not increased in patients with SSc, even in those with early disease despite the underlying vasculopathy; therefore, this particular pathway of platelet activation does not seem to be crucially involved in SSc pathogenesis.

### Clopidogrel treatment effectively inhibits ADP-dependent activation of platelets in patients with SSc

We next assessed the effect of clopidogrel on ADP-dependent activation of platelets in patients with SSc. Following 14 days of clopidogrel treatment a significant reduction of ADP-depended activation of platelets was found in patients with SSc (mean ± SEM AU*min: 392.1 ± 58.4 vs 163.8 ± 51.7, prior to and following treatment respectively, *p* = 0.014). These data are shown in Fig. 1b. This effect was sustained following one year of treatment (mean ± SEM AU*min: 157.2 ± 39, *p* = 0.005 compared to baseline). Two patients (15.4 %) showed resistance to clopidogrel inhibition and displayed high platelet reactivity (497 and 594 AU*min) despite treatment. These data show that clopidogrel is an effective inhibitor o ADP-dependent platelet activation in patients with SSc since the percentage of patients displaying resistance to clopidogrel inhibition (15.4 %) is even lower than that reported for the general population which is estimated to be up to 30 % [26, 27].

### Clopidogrel treatment does not affect serotonin levels

**Fig. 2:**
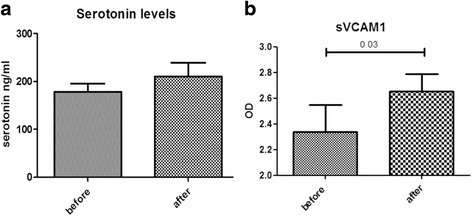
Effect of clopidogrel on serotonin and sVCAM1 levels. No change in serotonin levels in platelets poor plasma was found in patients with SSc following 14 days of treatment with clopidogrel (**a**). sVCAM1 levels are significantly increased following clopidogrel treatment in patients with SSc (**b**)

We next assessed whether effective suppression of ADP-dependent platelet activation by clopidogrel can decrease circulating serotonin levels, a significant profibrotic mediator. Serotonin levels in platelet poor plasma did not change following 14 days of treatment with clopidogrel (mean ± SEM ng/dl: 178.3 ± 17.4 vs 210.6 ± 28.6, prior to and following treatment respectively, *p* = 0.17), despite the effective suppression of ADP-dependent activation of platelets at the same time point. Data are shown in Fig. 2a.

### Clopidogrel treatment associates with worsening of endothelial function

We further evaluated the results of clopidogrel treatment on soluble VCAM1 levels as a marker of endothelial dysfunction/injury. There was a statistically significant increase of s-VCAM1 levels following 14 days of clopidogrel treatment (optical density mean ± SEM: 2.339 ± 0.211 vs 2.651 ± 0.139 before and following treatment respectively, *p* = 0.03) as shown in Fig. 2b. These data indicate that clopidogrel treatment does not improve but may actually worsen endothelial function in patients with SSc.

### Clopidogrel treatment may associate with the development of new digital ulcers in patients with SSc

Three patients, all with limited disease, developed new digital ulcers during the study; 2 of them did not have a history of digital ulcers. Patient #3 had early disease with a 3 year history of Raynaud’s phenomenon before the diagnosis, but no history of ulcers. Most striking, patient #13 had a longstanding disease of 15 years; she never had digital ulcers. Patient #4 had a 12 year history of disease but she has not developed any ulcers for the last 8 years, while on bosentan treatment. Patients #3, #4 and #13 developed the ulcers following 10, 9 and 10 months of treatment, respectively. In patients #13 and #3 we initiated treatment with bosentan with a good response after a short period, while patient #4 was treated with iloprost and antibiotics showing also a rapid improvement. None of the patients had any changes in everyday lifestyle during the study including exposure to cold. Following the development of digital ulcers in patient #13 the Safety Board of the study decided to withdraw all patients from the study medication, concluding that there may be an association between treatment and development of new ulcers. At that time point all patients, apart from patient #13, had completed 1 year on treatment. No other serious adverse events were recorded during the study. The main side effects included a transient rash after treatment initiation in patient #7 and minor bruising episodes in patients #5 and #9.

### Effect of Clopidogrel treatment on clinical outcomes

**Fig. 3:**
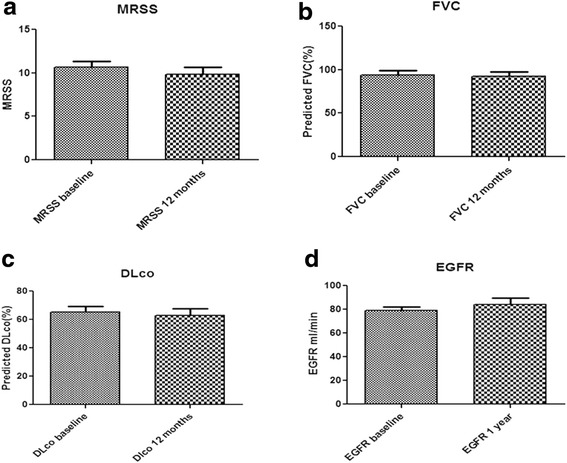
Effects of clopidogrel on clinical outcomes. Clopidogrel treatment does not have any significant impact on clinical outcomes (MRSS (**a**), FVC (**b**), DLco (**c**) and EGFR (**d**)) in patients with SSc

To evaluate any potential clinical effect of clopidogrel treatment we performed a complete physical exam, evaluation of skin thickening, evaluation of pulmonary function, basic laboratory work up and finally evaluation of general functional status. Clopidogrel treatment had no significant effect on skin thickening (mean ± SEM 10.69 ± 0.62 vs 9.85 ± 0.84 at baseline vs 1 year, respectively, *p* = 0.12). We did not detect any statistically significant differences in all indices of internal organ function we examined. FVC remained stable following 1 year of treatment (mean ± SEM 93.75 ± 5.58 vs 92.67 ± 5.04 at baseline vs 1 year, respectively, *p* = 0.69), the same was found for DLco (mean ± SEM 65.08 ± 4.31 vs 62.67 ± 4.85 at baseline vs 1 year respectively, *p* = 0.39). SHAQ also showed no significant change following one year of treatment with clopidogrel (mean ± SEM: 0.80 ± 0.11 vs 0.98 ± 0.14, *p* = 0.16). RVSP and EGFR also remained stable following 1 year of treatment. Data are diagrammatically shown in Fig. 3.

## Discussion

We report herein the results of an open label, proof of principle study evaluating the efficacy of clopidogrel in patients with SSc. So far, ADP-dependent platelet activation has not been investigated in SSc with newer techniques. The potential role of platelets in the pathogenesis of SSc has been explored in SSc but data are still controversial. Several studies demonstrate increased platelet activation using different agents as agonists [28, 29], but this was not a consisted finding [30].

In our study we found no evidence of increased ADP-dependent platelet activation in patients with SSc, even in those with early disease, compared to patients with RA and healthy subjects, indicating that this particular pathway of platelet activation may not be crucially involved in SSc pathogenesis. However, one should take into account that SSc is an extremely heterogeneous disease and therefore, definite conclusions cannot be drawn based on data derived from a small number of patients. One cannot exclude the possibility that there may be a subset of patients with SSc displaying increased ADP dependent platelet activation; we cannot rule out that in these particular patients clopidogrel might have some efficacy.

Moreover, we demonstrated that clopidogrel can effectively inhibit the ADP-dependent activation of platelets in the majority of SSc patients. Previous studies have reported that resistance to clopidogrel inhibition in particular populations, such as patients with chronic kidney failure under hemodialysis, can reach up to 84 % [31]. In our study only two of 13 SSc patients showed resistance to clopidogrel; this percentage (15.4 %) is even lower than that reported for the general population, which is estimated to be up to 30 % [26, 27].

Based on experimental data which indicate that platelets may be involved in the fibrotic process, we aimed at exploring whether platelet inhibition by clopidogrel could favorably affect fibrosis in patients with SSc, offering thus an adjunct therapeutic approach for the disease. There is evidence that serotonin is an important mediator in SSc which links vascular damage, platelets and fibrosis. Serotonin, a molecule stored in platelets, has been shown to possess profibrotic properties [32, 33]. However, there is no robust evidence that plasma free serotonin is increased in SSc. Biondi *et al* reported such a finding in patients with secondary Raynaud’s phenomenon [15], but Klimiuk *et al* found no evidence of this increase in patients with SSc [34]. In our study, effective inhibition of ADP dependent platelet activation with clopidogrel did not affect serotonin levels.

Finally, our data suggest that clopidogrel may associate with worsening of endothelial function and development of new digital ulcers in patients with SSc. Although platelets are considered to promote vasoconstriction, it is also known that they produce nitric oxide (NO) as well [35]. NO is a vasodilator which mainly derives from endothelial cells and consists an important modulator of vascular tone. It has been found that NO production from platelets is increased in chronic kidney failure [36], another condition characterized by generalized vasculopathy, and may have a protective role against cardiovascular risk. In addition, there are also studies demonstrating some protective features of platelets regarding fibrosis and vascular function. In two murine models of liver damage platelets seem to prevent fibrosis [37] and promote liver tissue repair [38]. Joshi *et al* showed that platelet depletion delays resolution of necrosis in the postischemic liver, suggesting that the presence of platelet-derived factors is necessary for tissue remodeling [37].

Moreover, Holowatz et al reported that platelet inhibition with clopidogrel attenuated reflex vasodilation, in middle aged women, suggesting platelet involvement in reflex vasodilation through the release of vasodilating factors. Investigators found that clopidogrel decreased skin blood flow responses during hyperthermia [39]. This effect may be of importance in SSc patients and may explain the development of new digital ulcers in our study. All the above data suggest that platelets under certain conditions may have properties which improve endothelial function and enhance vasodilation and tissue repair, rather than provoke tissue injury.

## Conclusions

Overall, our data indicate that clopidogrel may associate with worsening of endothelial function and development of new digital ulcers in patients with systemic sclerosis. This study in combination with previous reports of failure of antiplatelet agents in SSc, indicates that platelets may not play a crucial role in SSc pathophysiology, at least in late stages. Moreover, our data indicate that clopidogrel should be used cautiously in patients with SSc. This becomes even more important nowadays, as it is becoming clear that patients with SSc have increased cardiovascular morbidity and frequently need antiplatelet treatment [40]. Aspirin may be a more suitable therapeutic option for these patients. Our findings originate from a small uncontrolled trial and further investigation with larger cohorts is warranted.

### Ethics approval and consent to participate

A local (Patras University Hospital, Patras, Greece) Ethics Committee approved the study protocol (which fulfilled the Declaration of Helsinki requirements) and written informed consent was obtained from all participants.

### Consent for publication

Not applicable.

### Availability of data and materials

Experimental data are available in LabArchives repository https://mynotebook.labarchives.com/share_attachment/kostas/MTkuNXwxNzk0NjAvMTUtMi9UcmVlTm9kZS8zODM0NTIxMjM4fDQ5LjU=xMjM4fDQ5LjU=

## Abbreviations

SSc: Systemic Sclerosis
5-HT: 5-hydroxytryptamine
RA: rheumatoid arthritis
ADP: adenosine diphosphate
Anti-Scl-70: anti-topoisomerase I antibodies
anti-ACA: anti-centromere antibodies
PFTs: pulmonary function tests
FVC: forced vital capacity
DLco: diffusing capacity of carbon monoxide
RVSP: ventricular systolic pressure
eGFR: estimated glomerular filtration rate
MDRD: modification of diet in renal disease study group formula
SHAQ: scleroderma health assessment questionnaire
MRSS: modified Rodnan skin score
RIA: radioimmunoassay
s-VCAM1: soluble vascular cell adhesion protein 1
ELISA: enzyme-linked immunosorbent assay
